# High-performance HfO_*x*_/AlO_*y*_-based resistive switching memory cross-point array fabricated by atomic layer deposition

**DOI:** 10.1186/s11671-015-0738-1

**Published:** 2015-02-18

**Authors:** Zhe Chen, Feifei Zhang, Bing Chen, Yang Zheng, Bin Gao, Lifeng Liu, Xiaoyan Liu, Jinfeng Kang

**Affiliations:** Institute of Microelectronics, Peking University, #5 Yiheyuan Road, Beijing, 100871 China

**Keywords:** RRAM, Cross-point array, Atomic layer deposition (ALD)

## Abstract

Resistive switching memory cross-point arrays with TiN/HfO_*x*_/AlO_*y*_/Pt structure were fabricated. The bi-layered resistive switching films of 5-nm HfO_*x*_ and 3-nm AlO_*y*_ were deposited by atomic layer deposition (ALD). Excellent device performances such as low switching voltage, large resistance ratio, good cycle-to-cycle and device-to-device uniformity, and high yield were demonstrated in the fabricated 24 by 24 arrays. In addition, multi-level data storage capability and robust reliability characteristics were also presented. The achievements demonstrated the great potential of ALD-fabricated HfO_*x*_/AlO_*y*_ bi-layers for the application of next-generation nonvolatile memory.

## Background

Metal oxide-based resistive random access memory (RRAM) has been extensively studied as one of the most promising candidates for next-generation nonvolatile memory due to the great performances such as fast switching speed, low operating voltage, 3D integration, and good compatibility with CMOS fabrication processes [1-5]. For high-density integration of RRAM array, a cross-point structure with the smallest cell area of 4 *F*^2^ is needed [6,7]. However, the metal oxide-based RRAM devices usually have a large variability [8-10], which hinders application in industries. Thus, it is imperative to seek an effectively technical solution to minimize the variability of RRAM devices.

Various transitional metal oxides such as HfO_*x*_ [11-13], TaO_*x*_ [14-16], TiO_*x*_ [17-19], and ZrO_*x*_ [20-22] have been reported as resistive switching materials. Among them, HfO_*x*_ is a superior resistive switching material, which has stable electrical properties, good process repeatability, and small leakage current [23,24]. Based on a previous work [25], an additional buffer oxide layer of AlO_*y*_ which has a larger oxygen ion migration barrier (*E*_m_) will confine the switching in the active oxide, which can improve the uniformity in HfO_*x*_-based RRAM devices. Both physical vapor deposition (PVD) and atomic layer deposition (ALD) have been applied to fabricate resistive switching layers. Compared to PVD, the ALD technique has more advantages at constructing uniform, conformal, and ultrathin films, which is a central component for high-density and 3D integration.

In this paper, the bi-layered HfO_*x*_/AlO_*y*_ films are deposited by ALD as the resistive switching layer of cross-point RRAM array, which shows the precise control of the resistive switching layer in thickness, uniformity, and conformity. The fabricated TiN/HfO_*x*_/AlO_*y*_/Pt RRAM devices in the cross-point array show excellent performances including low operation voltage (+2/−2 V), sufficient resistance ratio (>10), smaller cycle-to-cycle and device-to-device variations, and high yield (>95%). Meanwhile, multi-level data storage capability, good direct current (DC) endurance (>1,000 cycles), and retention (>10^4^ s at 85°C) properties are demonstrated in the devices.

## Methods

**Figure 1:**
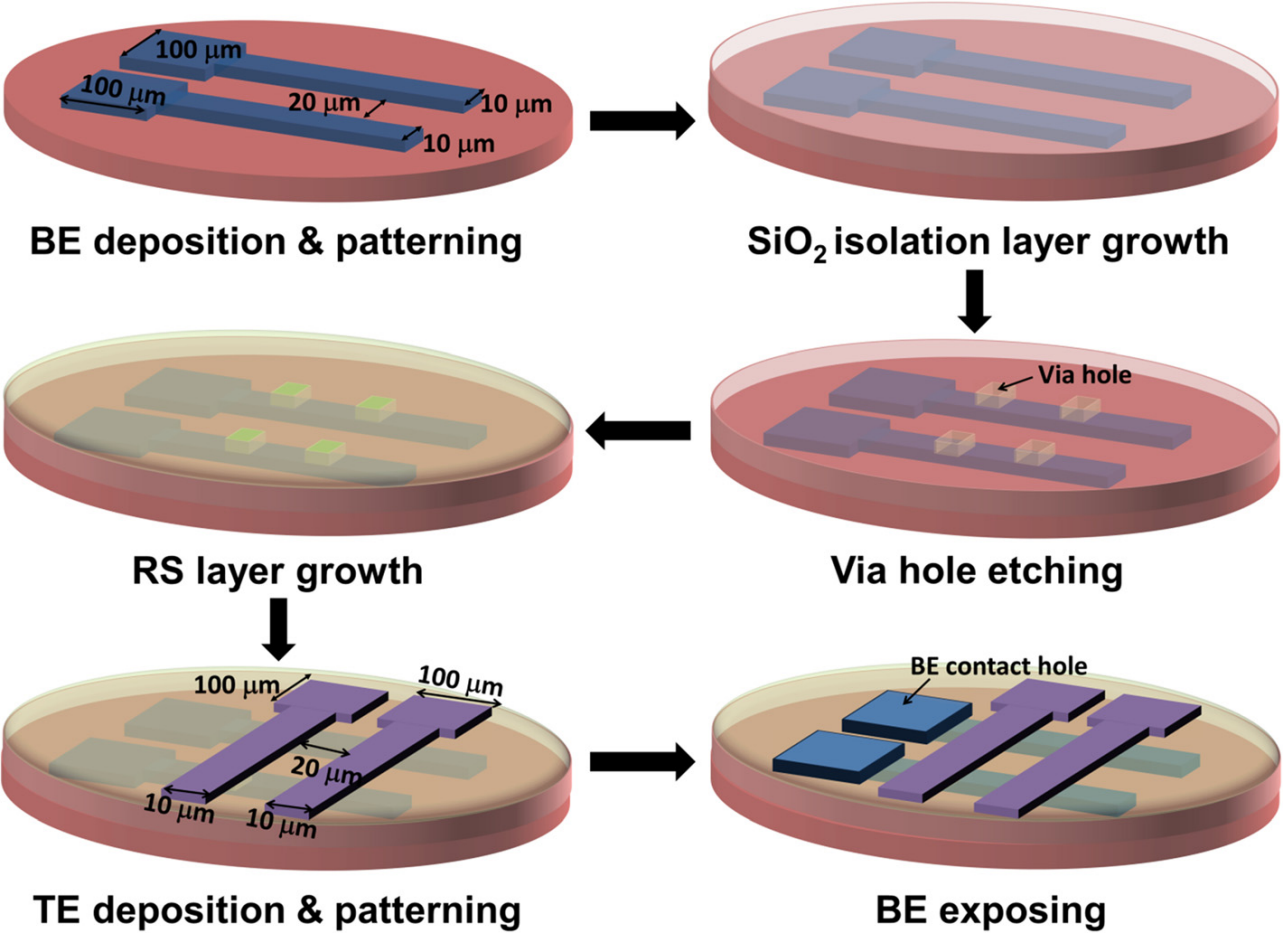
**Process flow of the fabrication of HfO**_*x*_ **/AlO**_*y*_ **-based cross-point RRAM array.**

The fabrication flow of the HfO_*x*_/AlO_*y*_-based cross-point RRAM array is schematically shown in Figure 1. Firstly, both the 20-nm Ti adhesion layer and 100-nm Pt bottom electrode (BE) layers were deposited on a SiO_2_/Si substrate by physical vapor deposition (PVD). Then, the Pt bottom electrode bars were formed by photolithography and lift-off. After that, the 20-nm SiO_2_ film was deposited by plasma-enhanced chemical vapor deposition (PECVD) to serve as the isolation layer. Different sizes of via holes through the SiO_2_ isolation layer from 1 × 1 μm^2^ to 10 × 10 μm^2^ were formed by reactive ion etching (RIE). Then, 3-nm AlO_*y*_ and 5-nm HfO_*x*_ layers were deposited by ALD (Picosun, Masala, Finland) at 300°C, using H_2_O and trimethylaluminum (TMA)/tetrakis[ethylmethylamino]hafnium (TEMAH) as precursors, followed by a furnace annealing in O_2_ ambient at 500°C for 30 min. After the 40-nm TiN was sputtered and patterned by photolithography and dry etching to define the top electrode (TE) bars, the contact holes to the pad of the bottom electrode Pt were formed by dry etching. The fabricated array size is 24 × 24, with cross-bar width of 10 μm and pitch along the *x* and *y* directions of 20 μm. The pad area of the electrodes is 100 × 100 μm^2^.

Electrical characterizations were performed using an Agilent B1500A semiconductor parameter analyzer (Agilent Technologies, Inc., Santa Clara, CA, USA). During the measurements, voltage was applied on the TE, while the BE was grounded.

## Results and discussion

**Figure 2:**
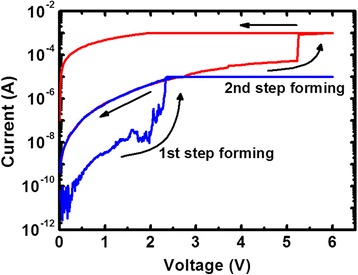
**Current–voltage curves of the two-step forming process.** The blue line is the first step, corresponding to the soft breakdown of the AlO_*y*_ layer, and the red line is the second step, referring to the soft breakdown of the HfO_*x*_ layer.

The resistance values of the fresh devices were usually higher than that of the high-resistance state (HRS) after a RESET process. A two-step forming process was required to activate the RRAM devices and achieve stable resistive switching behaviors. The current–voltage (*I*-*V*) curve of the forming process using voltage sweeping is shown in Figure 2. This two-step forming behavior can be attributed to the inhomogeneous distribution of the electric field in HfO_*x*_/AlO_*y*_ layers, which corresponds to the breakdown of HfO_*x*_ and AlO_*y*_ layers, respectively. The TE, resistive switching layer, and BE comprise a metal-insulator-metal (MIM) structure, which can be regarded as a plate capacitor with two kinds of dielectrics. According to Gauss’s law, when a voltage is applied across the TE and BE, the electric field intensity in the HfO_*x*_ layer and AlO_*y*_ layer can be obtained by the following equations:

1 $$ {\varepsilon}_{{\mathrm{HfO}}_x}{E}_{{\mathrm{HfO}}_x}={\varepsilon}_{{\mathrm{AlO}}_y}{E}_{{\mathrm{AlO}}_y} $$

2 $$ {E}_{{\mathrm{HfO}}_x}{d}_{{\mathrm{HfO}}_x}+{E}_{{\mathrm{AlO}}_y}{d}_{{\mathrm{AlO}}_y}=V $$

Here, *ε*HfO_*x*_/*ε*AlO_*y*_ refers to the dielectric constant of HfO_*x*_/AlO_*y*_, *E*HfO_*x*_/*E*AlO_*y*_ is the electric field intensity in the HfO_*x*_/AlO_*y*_ layer, *d*HfO_*x*_/*d*AlO_*y*_ is the thickness of the HfO_*x*_/AlO_*y*_ layer, and *V* is the value of the applied voltage. By calculating the above equations, the electric field intensity in the AlO_*y*_ layer is found to be stronger than that in the HfO_*x*_ layer. Therefore, the dielectric breakdown happens firstly in the AlO_*y*_ layer at a lower voltage, and then it happens in the HfO_*x*_ layer at a higher voltage.

**Figure 3:**
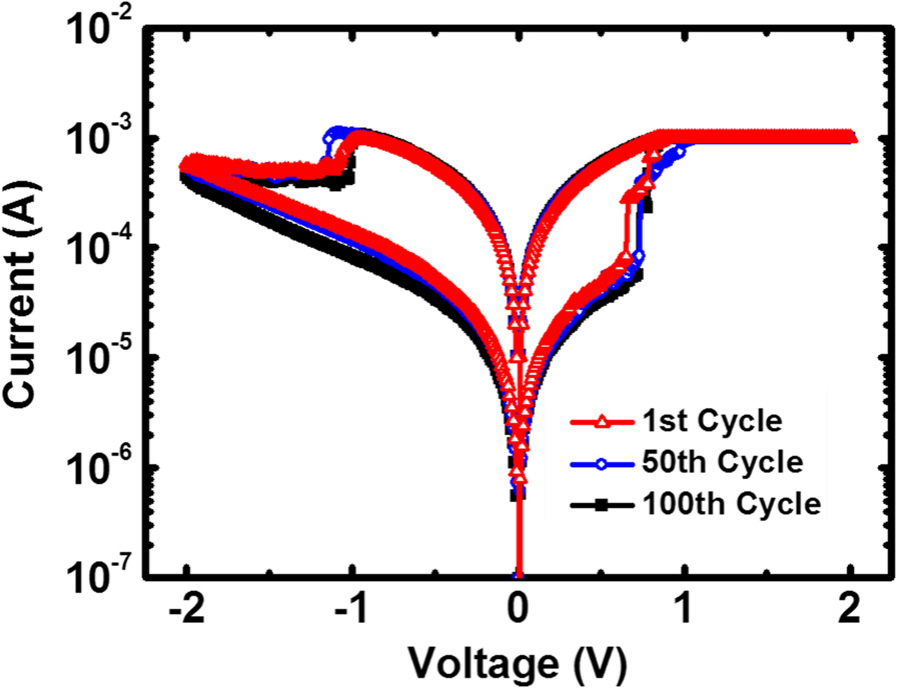
**Typical DC current–voltage curve.** Measured DC *I*-*V* characteristics of the HfO_*x*_/AlO_*y*_-based RRAM device for 100 consecutive cycles. Good cycle-to-cycle uniformity can be observed.

**Figure 4:**
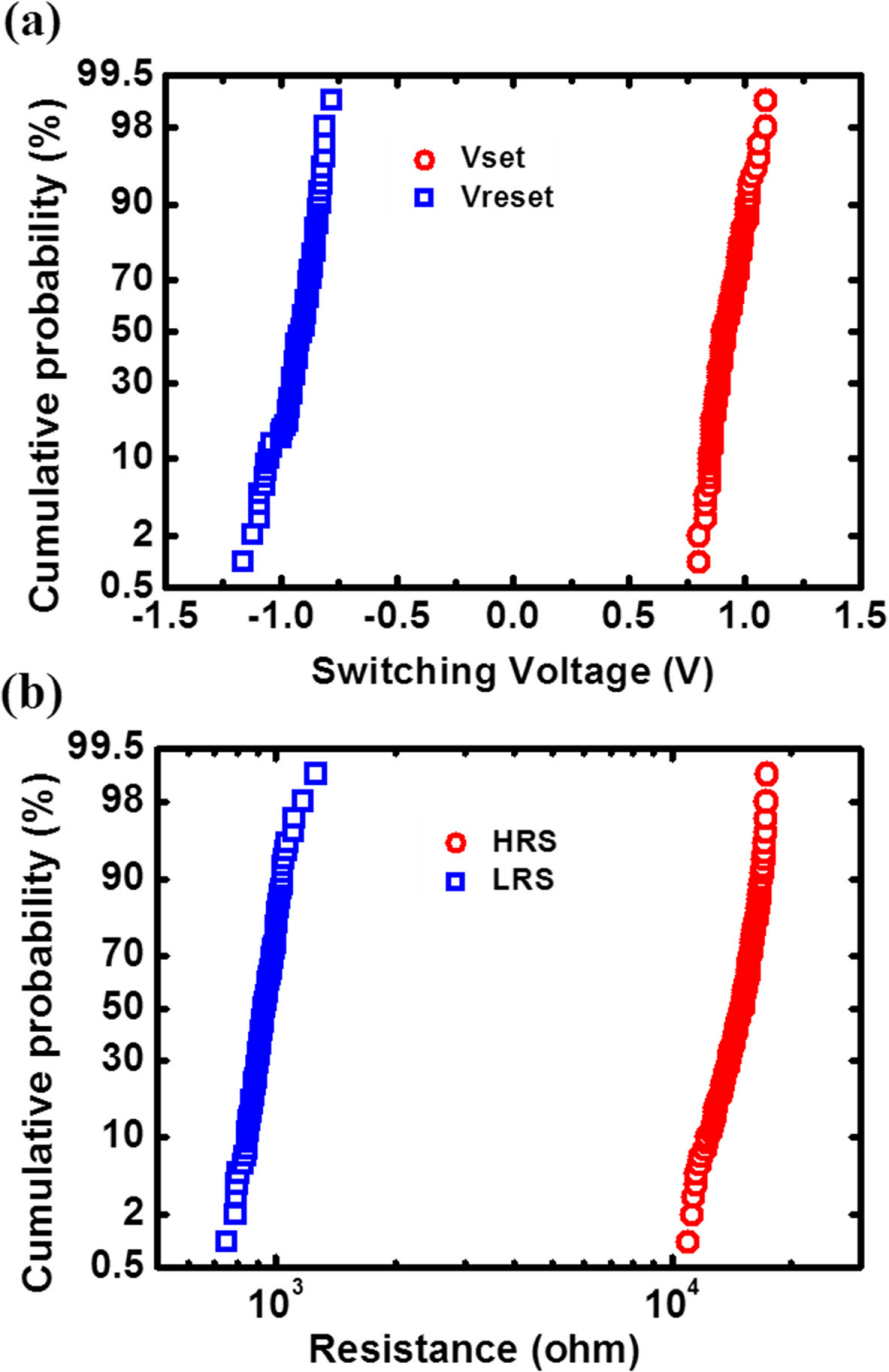
**Distributions of switching voltages and HRS/LRS resistances. (a)** Distribution of switching voltages. **(b)** Distribution of HRS and LRS extracted from the 100 consecutive cycles. The resistances were read at 0.1 V.

A typical DC *I*-*V* curve is shown in Figure 3. During SET/RESET operation, bias voltage was applied to the top electrode from 0 to +2/−2 V and then swept back to 0 V, while the bottom electrode was kept grounded. The devices show typical bipolar resistive switching behaviors, with the 1st/50th/100th DC *I*-*V* characteristics shown in the figure. The good consistency between the 1st, 50th, and 100th cycles reveals excellent switching cycle uniformity of the RRAM device. Moreover, both switching voltages and HRS/low-resistance state (LRS) distributions were obtained from 100 consecutive DC sweep cycles as shown in Figure 4a,b, respectively. In DC sweep mode, Vset means the voltage at which the current abruptly increases to the compliance current during the set process, and Vreset refers to the voltage at which the current begins decreasing during the reset process. The good cycle-to-cycle uniformity may be attributed to the interfacial effect of the HfO_*x*_/AlO_*y*_ layer [25]. The additional buffer oxide layer of AlO_*y*_ has a larger oxygen ion migration barrier (*E*_m_) and can confine the switching in the active oxide. Among the measured 150 uniformly distributed cells having one 24 × 24 array, only seven RRAM devices cannot switch, which shows the high yield (>95%) of the cross-point array.

**Figure 5:**
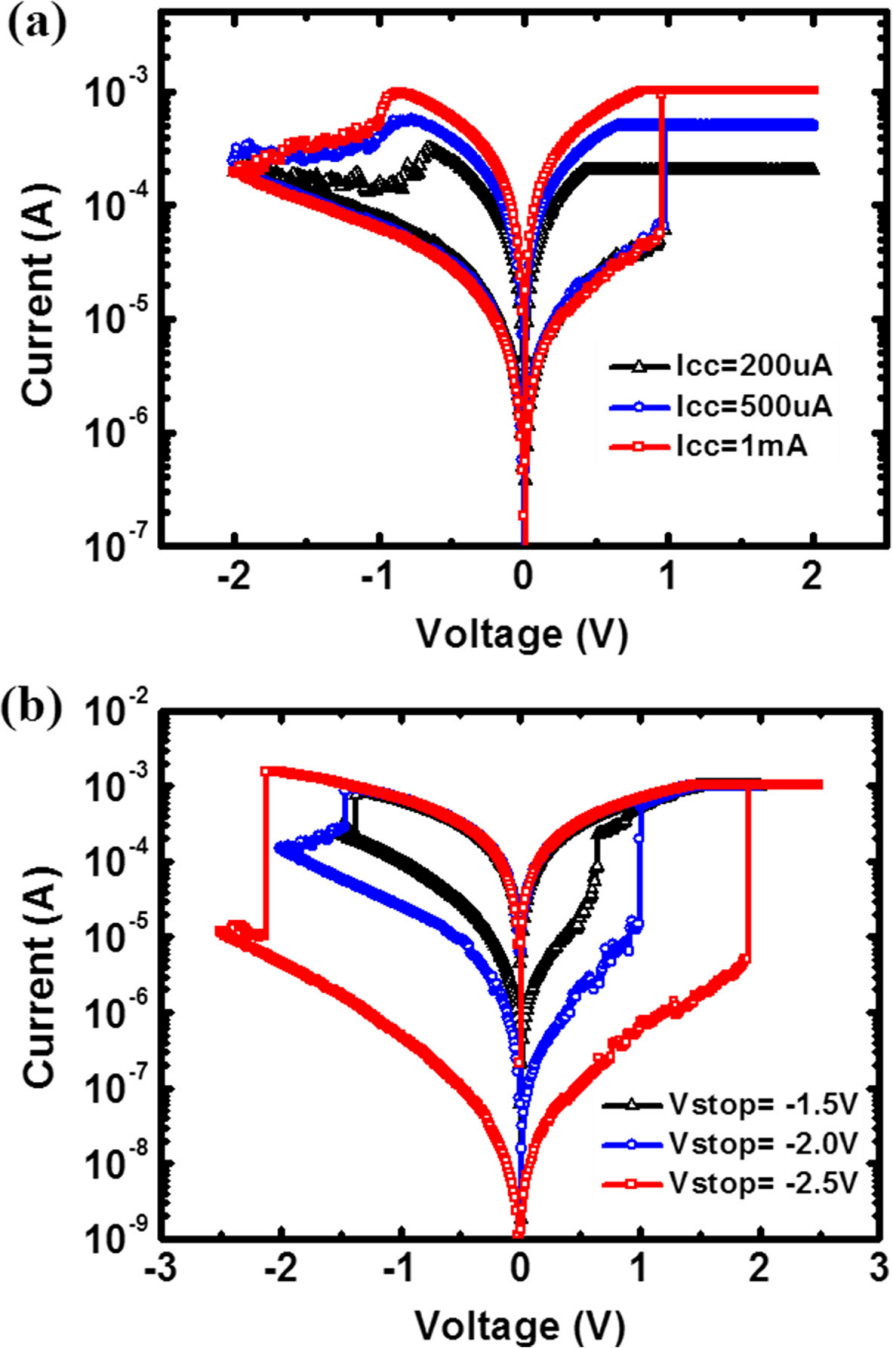
**Multi-level RRAM cell.** Multi-level resistance states achieved in the HfO_*x*_/AlO_*y*_-based RRAM **(a)** for the SET process by modulating current compliance, and **(b)** for the RESET process by modulating stop voltage.

A multi-level cell in RRAM is a desirable capability for high-density memory and neuromorphic computing system applications. The multi-level resistive switching behavior of the HfO_*x*_/AlO_*y*_-based devices can be achieved by adjusting both current compliance during the SET operation and stop voltage during the RESET process, as shown in Figure 5. The LRS resistance can be modulated by SET current compliance possibly due to the modulation of the diameter or number of conductive filament (CF), while the HRS resistance can be controlled by RESET stop voltage possibly due to the modulation of the ruptured CF length [24].

**Figure 6:**
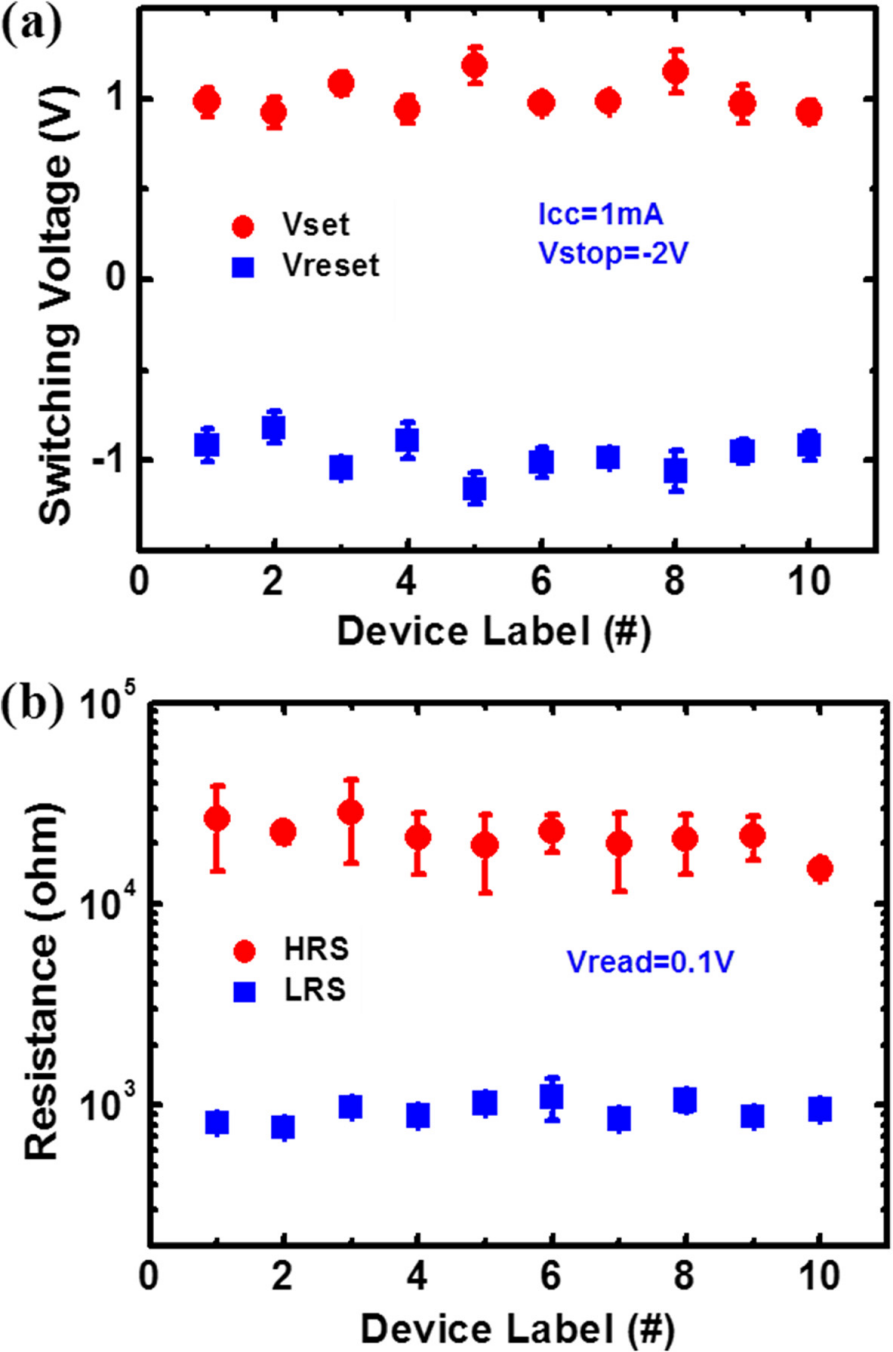
**Device-to-device variation. (a)** Measured device-to-device variation of HRS and LRS distributions. **(b)** Measured device-to-device variation of switching voltage distribution.

Excellent uniformity of the devices is crucial for array operation, since a large device-to-device variation of resistances or switching voltages may cause READ/WRITE failure. To investigate the device-to-device uniformity, resistance distribution and switching voltage distribution of ten devices were statistically measured and extracted. The results are shown in Figure 6a,b, with solid marks and error bars representing the mean values and standard deviations of 100 consecutive cycles, respectively. It can be found that the HfO_*x*_/AlO_*y*_ devices show good uniformity, which may be ascribed to the precisely controlled resistive switching layer properties in thickness, uniformity, and conformity of the HfO_*x*_/AlO_*y*_ layers by the ALD technique.

**Figure 7:**
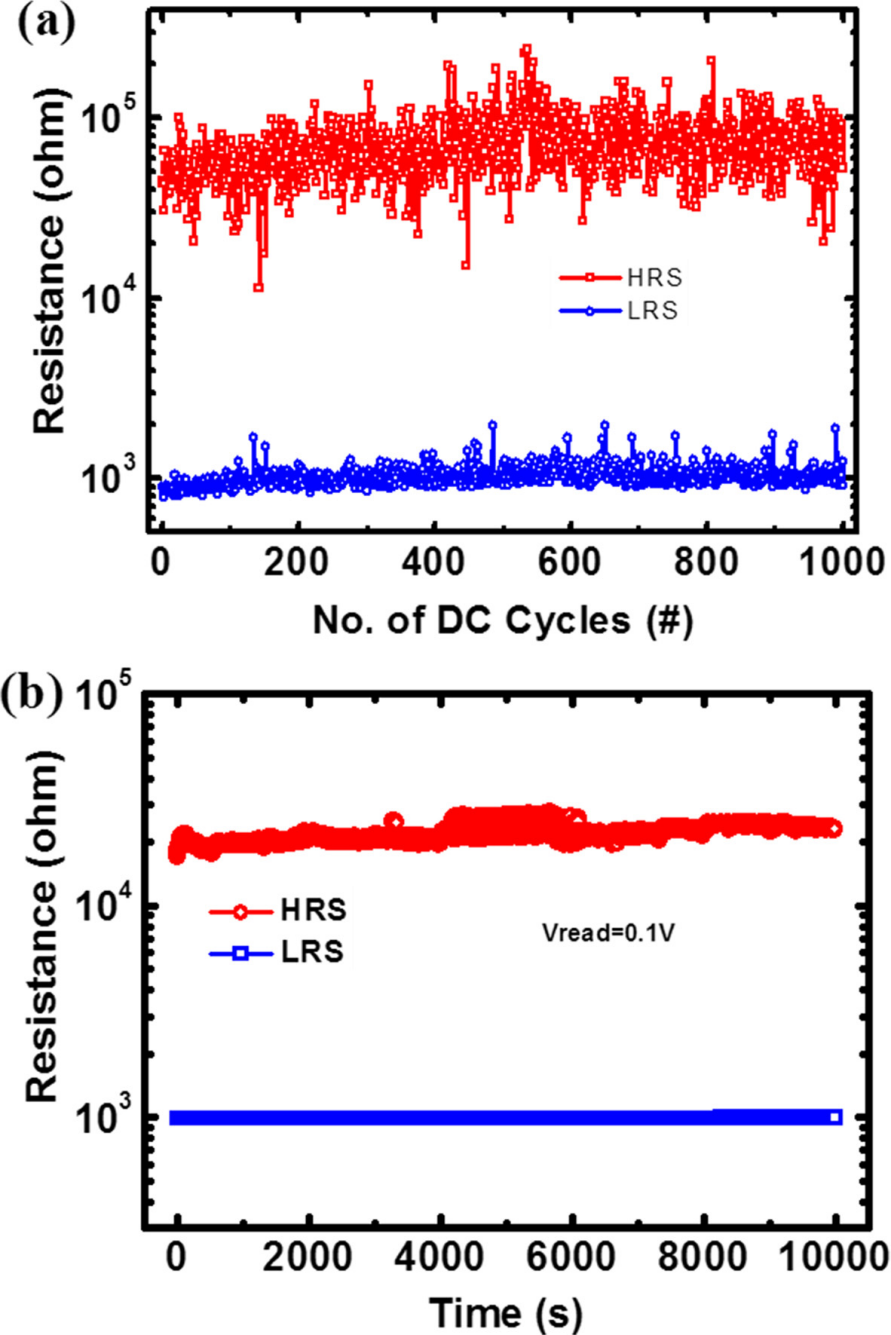
**Endurance and data retention. (a)** DC endurance characteristics for 1,000 cycles. **(b)** Data retention for both HRS and LRS for 10^4^ s at 85°C.

Figure 7a exhibits the SET/RESET endurance of 1,000 DC sweep cycles of the HfO_*x*_/AlO_*y*_-based RRAM devices. The set compliance current was 1 mA, and the reset stop voltage was −2 V. Both LRS and HRS were read at +0.1 V. Data of every cycle was extracted. Though the resistance is not very stable, the resistance ratio is always larger than 10.

In order to confirm the nonvolatility of the devices, time-dependent evolution of the resistance values of both HRS and LRS was monitored at 85°C. The resistance was read every second with a read voltage of 0.1 V. As shown in Figure 7b, both LRS and HRS show no signs of degradation for 10^4^ s.

## Conclusions

Excellent resistive switching characteristics of TiN/HfO_*x*_/AlO_*y*_/Pt RRAM devices in a cross-point array structure have been demonstrated in this work. The devices in the array show excellent cycle-to-cycle and device-to-device switching uniformity, which can be attributed to the precisely controlled HfO_*x*_/AlO_*y*_ bi-layered resistive switching layer by ALD and the effect on the resistive switching behaviors. These superior characteristics of the cross-point RRAM array could be useful for future nonvolatile memory applications.
